# Perceptions, practice, and “ownership:” experiences in continuity of the patient-doctor relationship in a family medicine residency

**Published:** 2017-12-15

**Authors:** Ann Lee, Sandra Kennett, Sheny Khera, Shelley Ross

**Affiliations:** 1 Department of Family Medicine, University of Alberta, Alberta, Canada; 2 Faculty of Nursing, University of Alberta, Alberta, Canada

## Abstract

**Background:**

The objective of this mixed-methods study was to determine interpersonal continuity (the ongoing therapeutic relationship between patient and health care provider) experiences of family medicine residents and preceptors, and explore their perceptions of interpersonal continuity.

**Methods:**

Quantitative data on resident and preceptor encounters were extracted from the electronic medical record (EMR). Opportunities for developing interpersonal continuity were determined using the Usual Provider Continuity (UPC) Index. A qualitative descriptive research method was used for the qualitative portion. Semi-structured interviews were conducted and constant comparative analysis was used to determine emerging themes.

**Results:**

Residents were found to have low UPC rates; preceptor rates were higher. Qualitative findings showed variable experiences with interpersonal continuity not apparent from UPC rates. Both preceptors and residents expressed perception of “ownership” of patients as a significant barrier to interpersonal continuity.

**Conclusion:**

This study suggests that a perceived lack of individual “ownership” of a patient panel was a significant barrier to developing interpersonal continuity. This might conflict with current changes towards team-based health care delivery. Understanding perceptions and changing them through a multi-faceted approach including resident teaching and faculty development might help improve interpersonal continuity which are core to both family medicine curricula and current models of health care delivery.

## Introduction

Family medicine residency programs across Canada aim to meet accreditation standards to ensure resident training is uniform and portable through the development of learning experiences that are consistent with the College of Family Physicians of Canada’s (CFPC) Triple C Competency-based Curriculum (Triple C).^1^–^6^ Triple C is a competency-based approach to family medicine education that was developed in response to the changing health care needs of the population. It is Comprehensive, focused on Continuity of education and patient care, and Centred in Family Medicine.^1^–^6^ Although voluntary, planning local curricula using the Triple C approach is key to providing quality education that prepares residents for their licensing examinations as well as their future practice in an evolving health care system.^2^

Paralleling the shift to the Triple C curriculum, change is occurring in the delivery of health care in Canada due to the increasing burden of chronic illnesses and rising health care costs within a fragmented system.^7^ Current research in primary care supports adoption of the patient-centred medical home (PCMH), a model of team-based care that is coordinated and sustained over time which is in contrast with traditional models where care is episodic and provided by individuals.^8^–^10^ Studies show that the PCMH “can improve appropriate service utilization and patient satisfaction,”^10^ but more research is needed to evaluate quality outcomes.

Central to both Triple C and PCMH is continuity of care.^1^–^6^,^8^–^10^ Although there is no consensus on the definition of continuity,^11^,^12^ Haggerty et al.^13^ attempted to develop a common understanding and defined continuity as “the degree to which a series of discrete health care events is experienced as coherent and connected and consistent with the patient’s medical needs and personal context.”^13^ Further, a report commissioned by the Canadian Health Services Research Foundation outlined three types of continuity: informational continuity; relational continuity; and management continuity.^7^ According to Haggerty et al.,^13^ relational continuity or the “ongoing therapeutic relationship between a patient and one or more providers” is “most valued in primary and mental health care.” The terms relational and interpersonal continuity of care are used interchangeably in the literature^14^,^15^ and Schultz stated “although all aspects of continuity of care are important, it is interpersonal continuity of care that is the strongest predictor of positive physician and patient outcomes.”^16^

Continuity of care is core to family medicine and residents are expected to experience this as outlined in the Triple C through the development and maintenance of a defined panel of patients or a list of individual patients under the care of a specific provider.^2^ However, Triple C recommendations are necessarily broad to allow for flexibility and innovation in residency training across Canada. As a result, some programs have adopted “half-day back” strategies where residents return to their primary teaching site for clinical sessions which are typically half a day or more each week in the hopes of increasing opportunities to develop continuity.^6^ Others have adopted a horizontal curriculum program structure where residents attend their teaching site throughout residency with other learning experiences occurring concurrently.^17^ The concept of continuity of care is also promoted in the PCMH, but it is one of continuity with a team rather than with an individual because in the PCMH, the focus is on care provided by multidisciplinary teams rather than by an individual.^9^ This may be perceived to be in conflict with the Triple C curriculum. Therefore, as primary care transforms towards PCMH as endorsed by the CFPC,^18^ it is increasingly important to understand the practice and perceptions of interpersonal continuity of care to meet the challenge of simultaneously providing good resident teaching and excellent primary care.^19^

There are limited studies focused on learner experiences with and perceptions of interpersonal continuity of care despite its importance to future practice. The objective of this mixed-methods study was to contribute to the current knowledge on interpersonal continuity of care among residents and preceptors in family medicine. This study uses both quantitative data from the electronic medical record (EMR) and qualitative data from individual interviews. The research questions for this study were:

1. What are the interpersonal continuity of care experiences of family medicine residents and their preceptors nearing the end of a 2-year residency?
2. How do family medicine residents and preceptors perceive interpersonal continuity with patients?

## Methods

### Design

A mixed-methods research design was used to study the interpersonal continuity of care experiences of residents nearing the end of a two-year family medicine residency program and their preceptors. The mixed-method approach was chosen because it provided a more complete picture of resident and preceptor experiences compared to the use of a single approach.^20^–^22^ We chose the triangulation model where qualitative and quantitative data were collected concurrently.^21^ Although the data were initially analyzed separately, the results and findings were used to confirm or reject inferences that arose from each approach and eventually integrated. The study was approved by the University of Alberta Health Research Ethics Board.

### Setting and participants

A purposive sample of graduating residents and their preceptors were recruited from a single Canadian academic family medicine teaching site located in a large urban city. During the study period from July 1, 2012 to June 30, 2014, there were six preceptors comprising a total clinical FTE of 3.3 and thirty first-and second-year residents who cared for a total of 4,816 active patients, all of whom are empaneled and have valid designated primary care providers. At this site, the main preceptor’s patient panel is considered to also be the resident panel. The program is organized into block time rotations. The core family medicine clinical experience consists of a six-month continuous block time during the first year and a once a week half-day back schedule where residents return for one half-day each week to their designated teaching site during the remainder of the program. All residents and their preceptors were given an information letter to invite them to participate. Preceptors who did not have graduating residents and residents not graduating at the end of the study period were excluded from this study because continuity experiences would be incomplete. Informed consent was obtained prior to individual interviews.

### Data collection

The EMR (MedAccess, Telus Health, Longueuil, Quebec) was adopted at the clinic in 2010. It allowed for retrospective data collection on preceptor patient panels and resident clinical encounters. Continuity of care rates were calculated using the UPC index which is the ratio of visits to the usual provider.^23^ Although the UPC Index provides a measure of repeat visits and is commonly used in studies measuring continuity in residency,^23^,^24^ it does not measure whether a therapeutic relationship was actually developed nor does it measure the quality of the relationship. However, multiple repeat visits are necessary in developing interpersonal continuity of care and the UPC Index provides a measure of opportunity for the development of interpersonal continuity.

### Statistical analysis

Descriptive statistics were used to summarize the quantitative data.

### Qualitative methods, subjects, and analysis

A qualitative descriptive approach was used to analyze the semi-structured interviews conducted by one of the researchers (SK).^25^,^26^ It is an approach that provides “rich, straight description” of experiences and “analysis that stays close to the data and the informants’ points of view.”^26^ Open-ended questions (Table 1) were used to explore experiences. Interviews of 11–29 minutes in length were audiotaped and professionally transcribed. Transcribed interviews were analyzed independently by two researchers (SK, AL) using ATLAS.ti 7 software^27^ and a constant comparative approach was used to develop an in vivo coding scheme which is a method of creating coding topics using terms taken directly from the participants’ own words.^28^,^29^ This type of coding allows for themes and ideas to stay close to the participant experience.^28^,^29^ The coding scheme along with reflective memos were then reviewed by two researchers (SK, AL). Discrepancies were discussed and a consensus was reached. To ensure rigor, member checking or participant validation through a presentation strategy of the preliminary results was used with some of the participants for their reaction and verification of findings.^30^

## Results

### Quantitative results

Of the five preceptors and six residents who met the inclusion criteria, all five preceptors (100%) and five residents (83%) participated in this study (Table 2). The one resident who was unable to participate in the interviews was on a rural rotation. There were a total of 2,412 patients seen by the residents. We excluded 462 patients who only had a single visit during the study period. Data were retrieved on 1,950 patients who presented to the clinic two or more times. The mean UPC Index for each resident ranged from 0.24 to 0.37 while the mean UPC Index for each preceptor ranged from 0.56 to 0.93 (Table 3).

### Qualitative findings

Participants identified interpersonal continuity as central to family medicine and central to the development of therapeutic alliances. It is why some participants chose family medicine and it is highly valued by all of the participants. In describing why it is central to family medicine, participants discussed the development of a therapeutic alliance with their patients, improved patient outcomes, and better learner experiences. Opportunities for interpersonal continuity allowed participants to understand the patients and work together with the patients to decide on the best approach for their health care needs.

Key to forming a therapeutic alliance is a sense of “ownership” on the part of the resident. “Ownership” and “own” were terms used by preceptors and residents. The preceptors identified “ownership” as an action that they would like to see in their residents. Although the preceptors wanted residents to take the initiative with the development of their patient lists, they recognized challenges associated with this expectation. The resident participants promoted the concept of their own clinic or a resident clinic and having patients booked for a clinic visit under the resident’s name, but were unsure about the feasibility of such a process. The residents felt that not only would these clinics improve opportunities for interpersonal continuity and the development of therapeutic alliances, but they would also motivate them to overcome some of the barriers to interpersonal continuity and relationship building.

Additional barriers to interpersonal continuity include patient factors such as their schedules or ability to return when a particular resident is available; preceptor and resident attitudes such as preceptor reluctance to give “ownership” and resident views on learning; and program factors such as curricular scheduling and competing expectations related to specialty rotations (see Table 4 in Appendix A). The participants frequently discussed scheduling as a barrier to continuity because despite efforts to encourage patients to return on a specific day and attempts at maintaining a fixed day for half-day back clinics, patients do not always follow-up with the same resident.

## Discussion

As residency programs align their curricula and learning experiences to meet accreditation standards that follow the Triple C curriculum, they are also challenged to integrate the concepts of the PCMH model of health care delivery. To adapt residency education to changes occurring in health care delivery, we need to understand current experiences and perceptions of interpersonal continuity of care among residents and preceptors. Previous studies have attempted to develop quantitative measures for understanding resident and preceptor experiences with interpersonal continuity, but there is no metric that sufficiently captures this.^31^ Though time intensive, interviews help explore experiences in more depth and provide additional understanding for the purpose of improving residency education.

In this study, we found continuity of care rates as measured using the UPC index were uniformly low among our residents and higher among the preceptors. This finding is not surprising as the preceptors have more clinical hours at the clinic. According to Francis et al., continuity for residents is significantly influenced by both numbers of clinics and panel size.^32^ In the literature, resident continuity is highly variable. One study reported resident continuity in ambulatory clinics ranging from 33.1% to 83.8%.^33^ While there is no agreed upon level of continuity that is considered appropriate in the primary care setting, previous studies have arbitrarily chosen goals between 0.50 and 0.70 using the UPC index based on panel size and number of clinics.^32^,^34^ Each of our residents had UPC rates below those reported in the literature and below suggested goals for resident continuity. The UPC index was useful in providing the basic understanding that patients were not seeing the same resident for the majority of their follow-up visits. However, it is difficult to make direct comparisons to existing literature given the differences in program structures and lengths of training. Although it is important to realize that quantitative data are valuable, qualitative data help to provide more understanding of interpersonal continuity experiences. From the qualitative data, we were able to explore variation in experiences despite uniformly low rates as well as reasons for the low continuity rates that have not been previously reported in the literature.

Even though all residents had low UPC indices suggesting limited opportunity in developing interpersonal continuity, interpersonal continuity experienced by the residents actually varied. This supports the idea that measuring quality rather than quantity of patient-resident interactions may be more meaningful.^31^ There were residents who felt they experienced interpersonal continuity during their residency and recalled them as positive experiences because they helped the resident learn about the illness process over time. Yet there were other residents who expressed difficulty with interpersonal continuity experiences particularly during half-day back clinics, which are meant to facilitate continuity. Despite varying continuity experiences, all participants felt interpersonal continuity was important.

The significance of developing interpersonal continuity as well as barriers to establishing interpersonal continuity have been reported in previous literature.^32^–^37^ Reviews have found significant positive relationships between interpersonal continuity and improved preventive care as well as reduced hospitalization.^35^ Reported barriers in the literature include “competing demands from in-hospital and subspecialty rotations as well as residency work hour restrictions.”^32^,^38^ In our study, similar barriers exist. However, the lack of control of how patients schedule their visits and program demands that take residents away from their scheduled clinics were barriers repeatedly identified by the participants. It is a challenge to restructure programs and there are limited reports on how to best structure and manage residency practices to foster continuity relationships.^34^ Despite this, we have recently consolidated aspects of the curriculum by introducing concentrated “Foundations” days where mandatory sessions are grouped into two full days rather than distributed as multiple half-days away from clinic throughout the year.

Another important finding in our study that is not described in the literature is the concept of “ownership” – a term repeatedly used by the participants. It is a term that seems paternalistic, but many participants identified “ownership” and related terms such as “my own” as being integral in the development of therapeutic alliances and interpersonal continuity. Preceptors commented that they would like to see residents take “ownership” of the patient care; however, they recognized their own reluctance to give up “ownership.” Residents voiced a desire for more “ownership” by proposing patients book follow-up appointments directly with them or creating a resident clinic. With “ownership,” one resident suggested there would be greater motivation to overcome interpersonal continuity barriers identified including scheduling challenges and demands of other rotations that lead to changes to resident half-day back clinics. The lack of “ownership” experienced by the residents was felt to contribute to the low continuity rates seen in this study.

This research supports that residents and preceptors continue to have traditional beliefs about individual “ownership” of a panel of patients in the development of interpersonal continuity. The challenge for medical educators is how to address the perception that having an individual panel separate from the preceptor panel is critical for the development of interpersonal continuity in care when health care delivery is moving towards the PCMH team-based model. Knowing this, however, will help curriculum developers explore options within the Triple C curriculum to provide clarity around expectations for continuity and integrate the curriculum with the PCMH model. This will also help medical educators consider ways to shift beliefs and perceptions that are barriers to developing interpersonal continuity through resident education and faculty development. For example, there needs to be a move away from the traditional belief of “ownership” to allow exploration of other ways of developing interpersonal continuity of care. As Greenzang states, the emphasis should not be “‘owning a patient,’ but rather owning the responsibility that comes with being someone’s physician.”^39^ Another way of addressing “ownership” as a barrier to continuity is teaching around how the PCMH model may offer opportunities for developing interpersonal continuity without the need for separate resident and preceptor panels. For example, a resident can become the most responsible physician for the duration of their residency while the preceptor remains an integral team member based on the PCMH model. This makes it possible to shift away from the perception that preceptor “ownership” and resident “ownership” are mutually exclusive in the development of a therapeutic relationship and move towards creating a team-based learning experience that is focused on shared responsibility rather than on individual “ownership” to align with the principles of both the Triple C curriculum and the PCMH model of health care delivery.

### Limitations and future directions

There are several limitations within this study. First, limitations inherent to mixed-methods research are present in this study including a sample size that is smaller than preferred for a quantitative study, and qualitative data focused on individual interviews rather than exploring external factors that influence resident experiences. As a result, we were unable to provide more advanced statistical analyses on additional factors that may be associated with continuity and we were unable to comment beyond participant experiences. Second, this study took place at a single urban academic site. It is an exploratory study and generalizability of results may be limited to similar teaching sites. Third, the focus of this study is on the interpersonal continuity experiences of residents and their preceptors while patient experiences were beyond the scope of this study. Patient experiences with and their perspectives on interpersonal continuity are equally important, but we must remember that relationships involve perspectives from all participants as medical educators strive to balance patient-centered care with learner-centered teaching.

Future research addressing the limitations of this study include a larger study that involves multiple sites (community and rural sites) to determine if the perceptions identified in this study occur in other settings as well as including perceptions from the patient perspective. We also encourage interpersonal continuity measurements in other residency programs for similar reasons and to identify programs that have been effective in promoting interpersonal continuity. Program changes made to try to increase opportunities for interpersonal continuity need to be evaluated to determine their effects on the patient-doctor relationship. Finally, this study raises questions about how perceptions of “ownership” may affect team-based care, which warrants further exploration.

### Conclusion

Our study provides resident and preceptor perspectives from the qualitative data and adds to insights from the quantitative data. This mixed-methods study identified that both residents and faculty perceive “ownership” to be a key component of developing interpersonal continuity. Curriculum developers need to provide clarity on expectations on interpersonal continuity in a changing health care system and educators must identify curricular mismatches with team-based models of care in order to integrate curriculum design with changes in health care delivery through understanding current learner experiences. In this case, instead of focusing on individual “ownership” in order for interpersonal continuity to occur, the PCMH may offer an opportunity to change this perspective and address challenges and barriers to the development of interpersonal continuity during residency.^40^

## Figures and Tables

**Table 1:** Interview questions

**The questions for the residents were:**	What is your understanding of interpersonal continuity in a family medicine practice? What has your experience been with interpersonal continuity during your time at the teaching clinic (includes 6-month block time and half-days back)? What changes would you make to improve interpersonal continuity with the patients you encounter?
**The questions for the preceptors were:**	What is your understanding of interpersonal continuity in a family medicine practice? What has your experience been with interpersonal continuity between your resident and your patients during the residents’ time at the teaching clinic (includes 6 month block time and half-day backs)? What strategies do you currently use to increase interpersonal continuity between your resident and your patients? What changes would you make to improve interpersonal continuity between your resident and your patients?

**Table 2:** Demographic characteristics of participants and patients, 2012–14

Resident	Resident Age	Resident’s Gender	Preceptors	Preceptor’s Gender	Preceptor Age	% Patients who were female	% Patients who were 65 or older
R001	28	Female	P002	Male	62	47	39
R002	28	Male	P001	Male	63	53	44
R003	27	Female	P003/4	Female/Female	39/43	81	21
R004	28	Female	P005	Female	46	63	28
R005	27	Male	P002	Male	62	42	42

**Table 3:** Clinical FTE and mean usual provider continuity index for each participant

Preceptor	cFTE	Mean UPC
P001	0.7	0.91
P002	0.6	0.89
P003	0.5	0.56
P004	0.3	0.65
P005	0.6	0.93

**Resident**	**cFTE**	**Mean UPC**

R001	0.2	0.32
R002	0.2	0.32
R003	0.2	0.26
R004	0.2	0.37
R005	0.2	0.24

cFTE, clinical full time equivalent

UPC, Usual Provider Index

## Appendix A

**Table 4:** Emergent themes from interviews with residents and preceptors about interpersonal continuity

*Theme*	*Exemplar Quotes*
**Interpersonal Continuity**	
Key to family medicine	“I think that is the attraction of family medicine.” P001 “I think it is important, that is why I chose family medicine. It is key to our specialty.” P003 “Those who choose family medicine choose it because you want to know what happened to that patient or what is going on. It is that story that you get to follow along.” P003 “I think that it is one of the largest benefits of family practice both for the patient and the physician and it is also at times one of the biggest challenges.” P005 “I guess being in family medicine, I think it is of utmost importance.” R003 “I think that following up with a patient on a regular basis helps you establish a therapeutic relationship as well which I think is one of the most important parts of being a family doctor.” R004
• **Therapeutic Alliance**	
Important for patient outcomes	“If you follow these other constructs which do not always agree with each other it means you have a relationship with a patient that allows you to make decisions in a way that you not otherwise have done, which improves their health care outcomes.” P002 “The reason you have continuity, the reason it is considered useful is that it improves patient outcomes.” P002 “It can have good benefits for both the practitioner as well as the patient to have someone who is seeing them from the start to the finish and help with their problem. It is a lot better than I think multiple different physicians trying to be involved and understand the same story or the same person over and over again.” R002 “I think especially during my training being in different areas, I really see how a lack of continuity of care actually affects patient care…So I guess when you know someone very well, you are taking care of them, you start to understand their way of thinking, their approach to their own health care which kind of helps direct you.” R003 “I also find it is helpful in providing care to patients because you are actually making decisions on patients you know and you have some idea of what their issues are…” R005
Important for education	“So the ability to follow up on whatever we started enables us to first of all assess is what we are doing working?” R001 “There are some memorable ones for sure where I started a work up for something that turned out to be a significant pathology and I think from a learning point of view but also from that relationship point of view I wanted to see how their care had been carried out up till that part. So there is certainly a vested interest and education interest as well but also from a relationship point of view that was important for me.” R001 “I think it helps if you have continuity because then you can see whether or not what you are doing actually works or not.” R005
• **Barriers**	
Ownership	“I think the way it is set up right now is that there is still a paternalistic thing; I am not sure how much ownership they feel for the patient versus the ownership that I feel for the patient.” P001 “How do we get the residents to take ownership of this, where the patient trusts them as their primary care giver rather than me as a preceptor.” P001 “The goal that I would love is residents to really feel like these are their patients and to build that relationship and to understand what that means in this type of teaching environment so that then later they can continue that on in whichever practice setting they chose to work in.” P003 “In a general sense, if you could improve ownership for the resident, then I think you would improve continuity, right?” P005 “I think it would be nice if the residents had their own clinic, so they would actually book with us but I know there are lots of logistical issues with that. But instead of booking with Dr. So and so, it would be with Dr. _. So it kind of almost feels like we are running our own show.” R003 “If there was a clean simple way where I could book them underneath my name and my schedule, then there would be more motivation for me to keep my half days back when I am supposed to be coming to you – because there is somebody specifically booked for me.” R004 “I think with a resident-based clinic, patients are booked with me rather than being booked with my preceptor so there would be a little bit more incentive for them to come in on days when I am available…I think it would be a bit more motivating just because then there are patients that are under my name and they are my patients rather than myself seeing my preceptor’s patient.” R005
Patient factors	“Logistically it does not work because the resident does not show up or the patient does not show up or they want to come on a different day.” P002 “I think almost all patients that come here realize that it is a teaching facility and they know that there is a resident and a lot of the patients are also picking up on the fact that the residents are building their own relationships with the patients and they are establishing that rapport as well.” R001 “I can be here the same day every week, but if we do not make an effort to schedule patients or tell the patients to come back on this day, then I am just here on a Tuesday and the patients are still random.” R002 “I mean I think my preceptor and I have tried very hard to kind of make sure that the patients I am following comes back on Tuesday mornings, but obviously that is very hard for some patients to always come on that day.” R003 “As we went to half days back, I had no continuity whatsoever because just the way that my schedule ended up being or the patient’s schedule just was not convenient to follow up every Tuesday morning or whenever my half day was.” R004
Preceptor and resident attitudes	“Perhaps we should be working on me at giving the residents their own list and being responsible for this. It is a hardship for me to make because I have spent so many years as me being in charge of that.” P001 “You think sometimes at the beginning you might pick patients who are going to be attached to one particular resident, but it does not necessarily work out that way depending on the patient’s schedule and the resident’s schedule but inevitably some always develops with certain patients and what the interest level of certain residents are and certain patients.” P004 “I think certainly in the way I teach I do not think I have necessarily done that to the degree that maybe other preceptors given their residents more freedom and I think that is only just because I am still quite selfish of wanting to take care of my own patients.” P005 “I do not feel that my education was sacrificed as a result of having a minimal amount of interpersonal continuity. You can look at it the other way that actually by seeing a more variety of patients for the first time you have a greater opportunity to practice your initial assessment and management of the initial visit rather than the follow up, which can often just be a diabetes check or seeing how their sugars are doing or how their leg is doing if you are thinking about cellulitis or something like that.” R002
Program factors	“Well, we try to book everybody on a Thursday and you back on a Thursday. Well, you can try to do that. Practically it does not work and even if you could make it work, how many patients need to come back within a six month block time, right?” P002 “I just feel like the continuity has dropped surprisingly since we went to 6 month blocks compare to 4 month blocks. The reason being is that the 6 month block is a bit moth-eaten now.” P004 “So certainly some positive experiences, but I think I missed out on some opportunities to just being physically absent from the site. The patients are coming in on days that I am not here.” R001 “I think that the process implemented is certainly an effective one, but there are certain limitations as I alluded to: physical absence and returning half a day a week often does not given enough of a return back to fully see the patient.” R001 “We did not have patients book on the day back. To be fair, it was kind of difficult because over the last year and a half, my day back has been all over the place because of our rotations and there is an unwritten standard that you are expected to be at the rotation at your time and you just try and find a day to come back where it does not mess with your rotation.” R002 “Definitely the six-month I felt that I kind of experienced that relational continuity the most just because you are here very day, you see those patients, you have that time where they can follow up. The half-day backs are a little bit more tough.” R003 “When the time comes, I actually can’t do my half day at the time that I was supposed to, so I have to reschedule it for a different time that week and then consequently I don’t get to follow up with the patient even though we had plans to do that. To be honest, a lot of the time when I am on something like orthopedics, surgery or internal medicine, I felt a lot of pressure to stay and finish the work.” R004
